# Immune Microenvironment of Muscular-Invasive Urothelial Carcinoma: The Link to Tumor Immune Cycle and Prognosis

**DOI:** 10.3390/cells11111802

**Published:** 2022-05-31

**Authors:** Oleksandr Stakhovskyi, Nazarii Kobyliak, Oleg Voylenko, Eduard Stakhovskyi, Roman Ponomarchuk, Oksana Sulaieva

**Affiliations:** 1 Department of Plastic and Reconstructive Oncourology, National Cancer Institute, 03022 Kyiv, Ukraine; stakhovsky9@ukr.net (O.S.); voylenko@ukr.net (O.V.); estakhovsky@ukr.net (E.S.); 2 Department of Endocrinology, Bogomolets National Medical University, 01601 Kyiv, Ukraine; 3 Medical Laboratory CSD, 03022 Kyiv, Ukraine; r.ponomarchuk@csd.com.ua (R.P.); o.sulaieva@csd.com.ua (O.S.)

**Keywords:** muscular-invasive urothelial carcinoma, tumor immune microenvironment, tumor-infiltrating lymphocytes, tumor-associated macrophages

## Abstract

In this study, we investigated the relationship between the tumor immune microenvironment (TIME), histological differentiation and hypoxia in patients with muscular-invasive urothelial carcinomas (MIUC) after radical cystectomy. Forty-two cases of pT2-3N0M0 MIUCs underwent clinical, histological and immunohistochemical evaluation by counting CD8+, FOXP3+, CD68+, CD163+ cells and polymorphonuclear leukocytes (PMN) in intra-tumoral and peritumoral areas, assessing PD-L1 and GLUT1 expression for defining the impact of tumor immune contexture on patients’ outcomes. Five-year survival rates and overall survival were calculated. Most of the MIUCs demonstrated the immune-desert or immune-excluded TIME, reflecting altered mechanisms of T-cells’ activation or traffic into tumors. Tumor immune contexture was closely related to histological differentiation. CD8+ cells were scant in MIUCs with papillary and squamous differentiation, while basal-like or mesenchymal-like histological differentiation was associated with increased density of CD8+ cells. A high rate of PD-L1 expression (47.6%) was not related to immune cell infiltration. M2-macrophages predominated under CD8+ lymphocytes. The abundance of PMN and CD163+ macrophages in MIUCs was associated with high GLUT1 expression. CD8+, CD68+, FOXP3+ cells and PD-L1 status did not affect patients’ outcomes, while high CD163+ density and PMN infiltration were associated with the unfavorable outcome of patients with MIUC. These data drive the hypothesis that in MIUC, immune escape mechanisms are shifted towards the role of the innate immunity cells rather than CD8+ lymphocytes’ functioning.

## 1. Introduction

Muscular-invasive urothelial carcinomas (MIUC) demonstrate a complex genetic landscape, substantial aggressiveness, poor treatment response and high death rate [1,2]. Nowadays, prognostication for bladder cancer is mainly based on clinical and pathological parameters [3,4]. However, MIUC progression and recurrence rates along with patients’ survival significantly vary under similar pathological staging and grading [5,6,7]. Discovering the genetic alterations underlying the pathogenesis of MIUC over the past decades has shed light on many aspects of MIUC pathogenesis [2,8,9,10]. The assessment of genetic and genomic profiles unlocked the sets of signatures allowing to define the molecular classification of MIUC [11,12]. Although these studies seem to be a breakthrough in understanding MIUC biology, the transcriptomic-based classification has not yet been translated to clinical practice due to some limitations [8,13].

Alternatively, a growing number of studies confirmed the clinical value of inflammation and immune cells for tumor prognosis and sensitivity to immunotherapy [14,15,16]. Tumor-infiltrating immune cells, including neutrophils, eosinophils, macrophages, dendritic cells and various subtypes of T-cells, are major contributors to the tumor immune microenvironment [17,18], affecting tumorigenesis and cancer progression. However, their prognostic significance in MIUC is under continuous evaluation [18]. Assessment of tumor-infiltrating CD8+ lymphocytes (TILs) number and spatial distribution is the core of the tumor immune cycle concept, allowing to distinguish which mechanisms of antitumor immunity are violated [19]. According to the tumor immune cycle concept, altered tumor neoantigens’ recognition, failure in T-lymphocytes’ priming, halted traffic of T-cytotoxic lymphocytes into the tumor or inhibited tumor cell killing define a variety of immune escape mechanisms in cancer [19]. Besides, it can provide prognostic information about a correlation between the number of TILs and the survival of patients with MIUC [20].

Nevertheless, the tumor immune cycle concept covers only the T-cytotoxic lymphocyte patterns, while other elements of tumor immune and metabolic contexture are out of focus. Evaluation of tumor immune contexture has revealed that most TIME cells were represented by tumor-associated macrophages (TAMs), polarized toward the anti-inflammatory M2 type, that facilitates cancer cells’ growth, motility and angiogenesis [21]. Other immunosuppressive cells, including neutrophils (TANs) and T-regulatory cells (Treg), have also been demonstrated to facilitate cancer progression and metastases of bladder cancer [22,23,24]. Intriguingly, tumors with various histological patterns possess different immunogenicity. The molecular classification of MIUCs also highlights the relation of distinct molecular subtypes to histological patterns and immune signatures. However, as yet, there is no clear understanding of the spatial distribution of immune cells and their interplay with cancer cells of various differentiation. Therefore, assessment of the balance between tumor-antagonizing mechanisms and immunosuppressive cells in MIUC of various histological types can uncover tumor–immune interplay and its impact on patients’ prognosis [15,25,26].

Tumor–host immunity crosstalk is highly dependent on local conditions, including hypoxia. Hypoxia fuels tumor invasiveness and genomic instability through the oxidative stress and the Warburg effect [27,28,29]. Hypoxic markers were shown to be an independent prognostic factor in surgically treated bladder cancer [30]. Besides, hypoxia impairs anticancer immunity by metabolic reprogramming of TIME cells [30]. It was shown that hypoxia shifts the tumor–immune interplay towards a cold and immunosuppressive microenvironment [21]. In this study, we investigated the prognostic value of both tumor-antagonizing and immunosuppressive cells in MIUC through the concept of the tumor immune cycle with respect to the histological type of MIUC and hypoxia.

## 2. Materials and Methods

### 2.1. Case Selection and Patient Characteristics

This is a retrospective study including 254 cases of patients with primary bladder cancer (aged 56.7 ± 9.6 years), who underwent cystectomy at the National Cancer Institute (Ukraine) from 2009 until 2014, with a 5-year follow-up. There were 221 (87%) males and 33 (13%) females. Major treatment was radical cystectomy that included surgical bladder removal of prostate (males), uterus and ovaries (females), that was performed with standard pelvic lymphadenectomy. Cases with neoadjuvant or adjuvant chemo- or radio-therapy were excluded from the study (*n* = 25). Additionally, we excluded patients with metastases to lymph nodes or distant metastatic disease (*n* = 112), as well as patients with other malignancies in their medical history (*n* = 12). To prevent the impact of confounding factors on overall survival (OS), we additionally excluded patients with upper urinary tract pathologies (hydronephrosis grade III–IV, *n* = 17), heart failure (NYHA 3–4, *n* = 9) and patients with transrectal urine derivations: ileosigmoid reservoir (*n* = 2) and Maints II method (*n* = 3). Since the prevalence of urothelial cancer is three times as high in males than females [31], and taking into account the prognostic significance of sex in bladder cancer, only males were enrolled for further investigation. Only cases with the full set of clinical and histopathological data, confirming MIUC (pT2-3) and with a complete follow-up period (*n* = 42) were selected for further immunohistochemical evaluation.

Clinicopathological variables for each case were recorded, and five-year survival rates, progression-free survival (PFS) and overall survival (OS) at last follow-up were calculated.

This study was approved by the Institutional Review Board (IRB) of the National Institute of Cancer (Protocol # 74 from 22 September 2015) and was performed in accordance with the principles of the Declaration of Helsinki. Informed consent was waived by the IRB.

### 2.2. Tissue Processing and Immunohistochemistry

The tissues taken after surgery were fixed in 10% neutral buffered formalin and processed. For each tumor, the histological subtype, divergence of differentiation, carcinoma in situ (CIS), lymphovascular invasion and tertiary lymphoid structures were assessed. In case of histological heterogeneity of tumors, the types of additional differentiation were recorded. The intensity of inflammatory infiltration by neutrophils and eosinophils was assessed using semiquantitative scores (0 to 3, as absent, mild, moderate and severe, respectively). Besides, the rates of mitosis and entosis features were considered. For immune cells, immunohistochemistry (IHC) was applied.

For IHC, serial sections of 4 μm in thickness were used. Tissues were deparaffinized and hydrated. Endogenous peroxidase activity was blocked using 3% methanol in hydrogen peroxide. Next, antigen retrieval in a water bath at 98 °C was performed using Tris EDTA or citrate buffer (pH 6), followed by incubation with primary antibodies. After washing, labeled polymer secondary antibodies (Envision Detection System, Dako, Agilent, Santa Clara, CA, USA) were added to the slides. Peroxidase activity was detected using diaminobenzidine (DAB)-tetrahydrochloride liquid plus a Chromogen System (Dako, Agilent, Santa Clara, CA, USA) substrate. The reaction was stopped with distilled water. After that, sections were counterstained with hematoxylin and mounted in Richard-Allan Scientific Mounting Medium (Thermo Fisher, Waltham, MA, USA).

To visualize T-cytotoxic lymphocytes that are effector cells of cell-mediated antitumor immunity, we used antibodies to CD8 (Dako; Clone C8/144B). FOXP3 (Cell Marque, Clone EP340) was used for the defining T-regulatory cells. CD68 (DAKO, Clone KP1) and CD163 (Cell Marque, Clone MRQ-26) were used to visualize macrophages of different phenotypes. Additionally, PD-L1 (Dako, 22C3) expression was assessed in all the selected cases for uncovering tumor immune escape mechanisms. For assessing the impact of hypoxia on the immune contexture, the evaluation of GLUT1 (polyclonal, Cell Marque, Rocklin, CA, USA) expression was performed immunohistochemically (Table 1). 

When assessing different immune cells, we examined their number and spatial distribution with respect to tumor heterogeneity. The density of CD8+, FOXP3, CD68 and CD163+ cells was counted within tumor nests (TN) and in the peritumoral stroma (TS) in 10 visual fields corresponding to “hot spots”, with further quantification per 1 mm^2^. In tumors with variable histology, every component of certain histological differentiation was evaluated separately for assessing the links between morphological patterns and tumor immunogenicity. The primary histopathological evaluation was performed microscopically by two independent pathologists. Besides, digital images of sections were captured using a digital slide scanner (3DHISTECH, Budapest, Hungary) and assessed in a blind fashion. 

The number of immuno-positive cells was assessed as both continuous and dichotomized variables using cut-off values (52 cells per mm^2^ as a median for CD8+ cells, 27 for CD68+ cells and 86 for macrophages) to discern low-density from high-density results. As FOXP3 cells were scant, tumors containing >3 FOXP3 cells were considered as FOXP3_high_. Immune cells in vessels, submucosal lymphatic areas and areas adjacent to necrosis were not counted in this study. The expression of PD-L1 was evaluated using the combined positive score (CPS) according to the percentage of stained tumor cells or immune cells (lymphocytes and macrophages) in proportion to the total number of tumor cells. It was defined as positive if CPS ≥ 1%. Only membranous staining was considered as positive when assessing PD-L1 expression, regardless of the intensity. GLUT1 expression was judged as positive in cases of membranous staining. Erythrocytes served as an internal positive control. For grading GLUT1 expression, the semiquantitative immunohistochemical score (range 0–12) was used. It was calculated as a multiplication of the intensity of staining (0—no staining, 1—weak, 2—moderate and 3—strong) and the percentage of immunoreactive cells. The percentage of positive cells was categorized as: 0 (≤5%), 1 (6–25%), 2 (26–50%), 3 (51–75%) and 4 (>75%) [37].

### 2.3. Methodology of Tumor–Host Immunity Assessment

For interpreting tumor–host interplay and mechanisms of immune escape, we assessed TIME according to the immune cycle concept. The evaluation of TIME in every case was performed for the predominating histological type of the tumor. In case of tumor heterogeneity, the histological type with worse prognosis was considered for assessing the TIME. The following types of TIME were considered: immune-desert (ID), immune-excluded (IE) and inflamed (Inf) [38]. The ID type reflects a lack of pre-existing immunity and demonstrates a few T-cells inside and around the tumor. The IE type shows prominent peritumoral infiltration but a few intra-tumoral T-lymphocytes because of failed T-cell traffic. The Inf TIME possesses high lymphocyte infiltration, reflecting the activation of antitumor T-cells with improper functioning [38].

### 2.4. Statistical Analysis

The descriptive statistics included counts and frequencies for categorical variables and the mean ± standard deviation (SD), with 95% confidence interval (CI) for continuous variables. Normally distributed continuous variables were analyzed with unpaired sample *t*-tests. When comparing continuous variables between 3 or more subgroups, the ANOVA test was used. Non-normally distributed continuous variables were assessed with non-parametric Mann–Whitney U-tests. The chi-square or Fisher’s exact test was used to compare categorical variables. The Cox regression and Kaplan–Meier analyses were performed with disease-specific survival as the endpoint. The statistical assessment of data was carried out using GraphPad Prism 7 software (GraphPad, San Diego, CA, USA). A *p*-value of <0.05 was considered statistically significant.

## 3. Results

Amongst 42 male patients with muscular-invasive urothelial carcinoma, there were 30 cases with pT2 (71.4%) and 12 (28.6%) with pT3 (Table 2). The 5-year disease-specific survival comprised 69%, while the PFS rate was 47.6%. Twenty-two patients (52.4%) developed local/regional recurrences or distant metastasis. 

OS comprised 52.2 ± 22.2 (95% CI 43.9–60.5) months among pT2 cases and 35.8 ± 27.3 (95% CI 18.4–53.1) for patients with pT3 (*p* = 0.049). Five-year survival rate comprised 77.7% and 50% in patients with pT2 and PT3, respectively.

The assessment of the histological features of MIUC revealed 14 (33.3%) cases with CIS, 22 (52.4%) tumors with lymphovascular invasion (LVI) and 26 (69%) cancers with prominent tumor heterogeneity and variant histology. Not surprisingly, these factors affected patients’ prognoses (Table 3).

**Figure 1:**
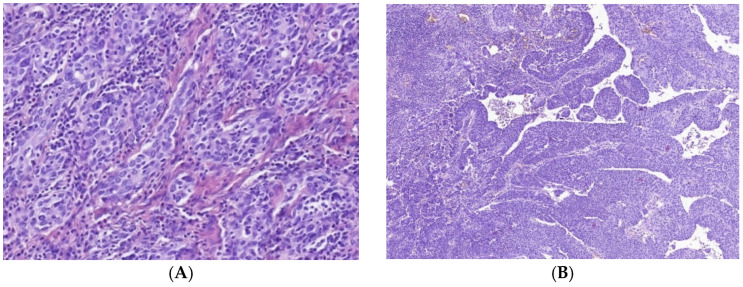

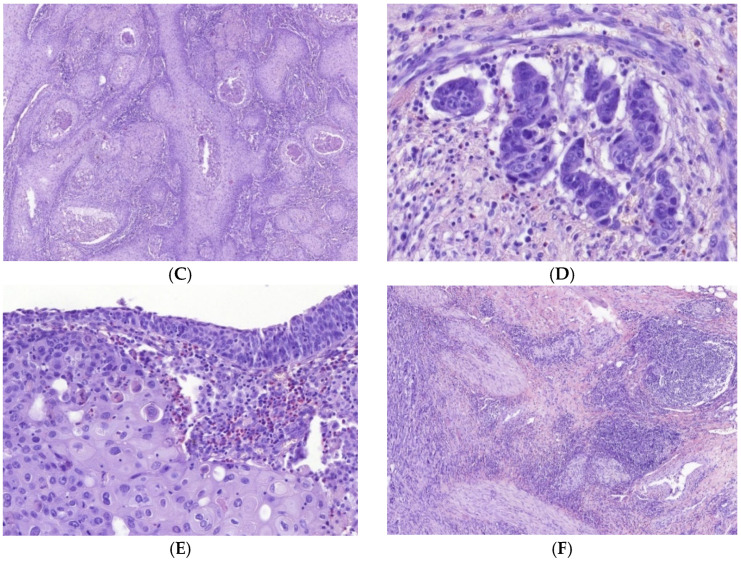
Histological heterogeneity of MIUCs. In addition to conventional histological patterns (**A**), muscular-invasive urothelial carcinomas yielded papillary (**B**), micropapillary (**D**), squamous cell (**C**) differentiation and other types of divergent differentiation. Besides, MIUCs varied in the density of inflammatory infiltration by innate and adaptive immunity cells (**E**,**F**). Some cases possessed CIS, mostly in complex with squamous cell differentiation, while others had tertiary lymphoid structures within the bladder wall. Hematoxylin and eosin staining. Magnification: (**A**,**E**) 200, (**B**,**C**,**F**) 100, (**D**) 400.

MIUCs demonstrated high variability and heterogeneity of histological differentiation. There were 26 cases with areas of variant histological differentiation (Table 4, Figure 1). Different histological patterns were related to specific immune cell sets, number and distribution. Among tumors with uniform morphology, there were nine cases with papillary patterns and seven carcinomas with the conventional structure of urothelial-invasive carcinoma (UIC). The rest demonstrated significant heterogeneity by combining conventional UIC with divergent differentiation (Table 4), and four cases (10%) displayed more than two histological variants.

### 3.1. Immune Cell Count and Distribution in MIUCs

**Figure 2:**
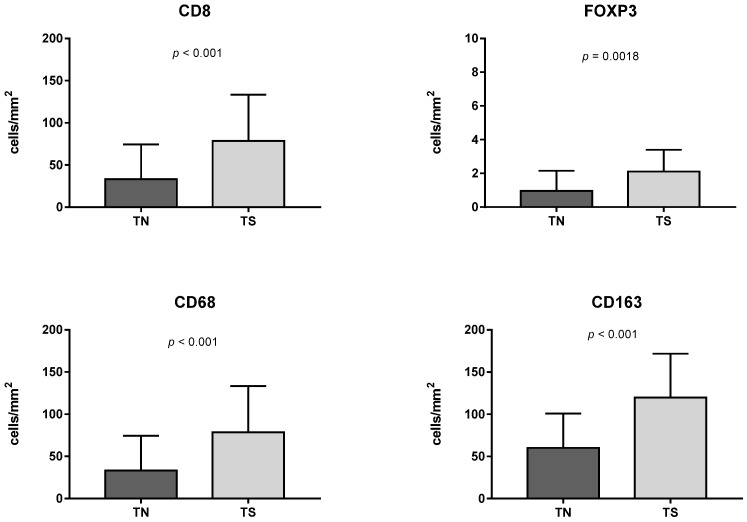
The number of immune cells in tumor nests (TN) and stroma (TS). Both lymphocytes and macrophages prevailed in the stromal compartment. Only scant FOXP3 cells were identified in MIUCs. The number of macrophages was significantly higher when compared to lymphocytes in both tumor nests and tumor stroma.

The number of CD8+, FOXP3+, CD68+, CD163+ and PMNs was highly variable, however, all immune cells prevailed in stroma when compared to tumor nests (Figure 2). FOXP3 cells were the fewest, while M2-type macrophages predominated under CD8+ lymphocytes. Surprisingly, CD68+ cells were less numerous than CD163+ cells, though CD68 is considered a pan-macrophages marker. Moreover, some tumor cells demonstrated positive staining for CD68, so this marker was excluded from the following analysis.

**Figure 3:**
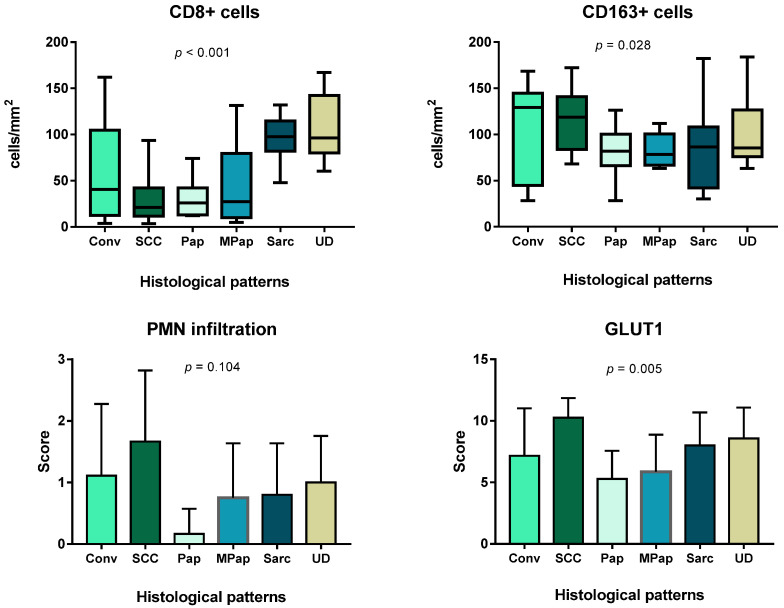
The immunogenicity of MIUC correlates with histological differentiation.

**Figure 4:**
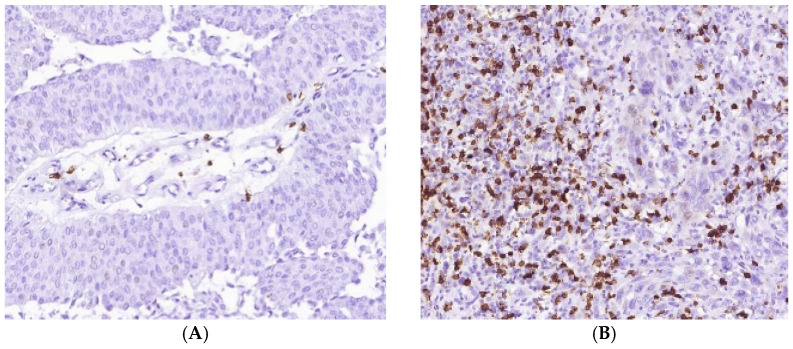

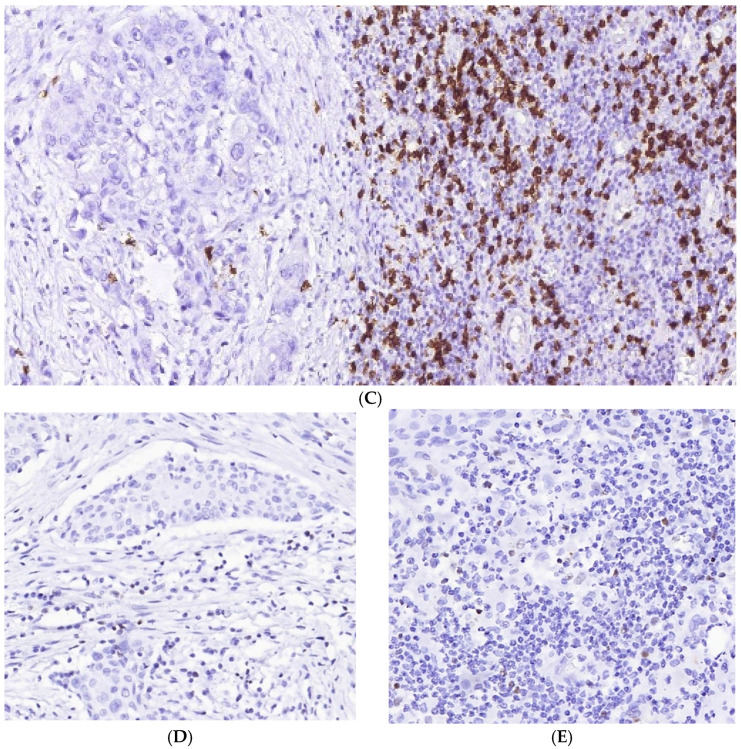
CD8+ and FOXP3 cells in MIUCs. The number of CD8+ cells varied in MIUCS of different histological subtypes. Most MIUCs with the papillary (**A**) and squamous ((**C**), left part) patterns were CD8+“cold”. MIUCs with conventional (**B**) or basaloid ((**C**), right part) yielded a “hot” immunophenotype. The prominent intra-tumor heterogeneity of immune contexture (**C**) was closely related to the histological differentiation of tumor cells. At the same time, FOXP3 cells were scant in MIUCs (**D**), but more numerous in areas of squamous differentiation (**E**).

MIUCs with a papillary pattern possessed the lowest inflammatory infiltration by PMN and CD8+ cells (Figure 3 and Figure 4). CD8+ lymphocytes were more numerous in urothelial-invasive carcinomas with conventional, basal-like and sarcomatous differentiation (*p* < 0.001). Lymphocytes also accumulated at the invasive margin of the tumor. Besides, tertial lymphoid structures were found in 15 cases that appeared as lymphoid follicles between muscle bundles in the bladder wall (Figure 2). However, they did not affect patients’ prognosis (*p* = 0.242), and there was no impact of CD8+ TILs and TIME on patients’ outcomes (Table 3).

The papillary pattern of MIUCs (Pap) was associated with low infiltration by CD8+ cells and PMNs. Areas with squamous cell differentiation yielded low CD8+ cells and demonstrated an abundance of CD163 and PNM. The highest rate of CD8+ cells was found in areas with basaloid morphology and in zones of mesenchyme-like (sarcomatoid) patterns. Besides, the papillary pattern was associated with the lowest score of GLUT1 expression, while the highest figures were found in zones of squamous differentiation.

Assessing Treg cells, we found only scant FOXP3 cells in the stroma of MIUCs, though their number was higher in areas with squamous differentiation and under CIS. At the same time, squamous cell differentiation was associated with predominant PMN infiltration, a high number of CD163+ cells and few CD8+ cells (Figure 4).

**Figure 5:**
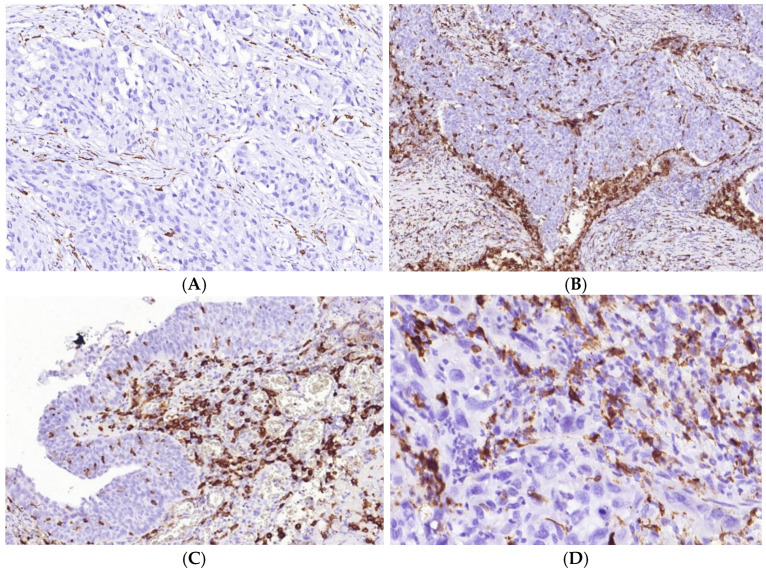
M2-macrophages’ number and distribution in MIUCs. There were MIUCs with low (**A**) and high (**B**) CD163+ cells. Numerous M2-macrophages accumulated under carcinoma in situ areas (**C**). Additionally, a high number of CD163+ cells co-occurred with severe PMN infiltration (**D**).

M2-macrophages formed a dense meshwork within and around tumor clusters in most cases. Besides, numerous CD163+ cells infiltrated lamina propria under CIS (Figure 5). The high density of CD163+ cells was found in areas with squamous, basaloid and sarcomatoid differentiation, though there were no statistically significant differences with other histological subtypes of MIUCs. 

PMN infiltration was the highest in areas of squamous differentiation as compared to other histological differentiation (Figure 1C,E). Interestingly, PMN density, but not number of CD8+ cells, positively correlated with GLUT1 expression (Figure 3) and negatively affected patients’ outcomes (p < 0.001).

### 3.2. Immune Cycle Patterns in MIUCs

**Figure 6:**
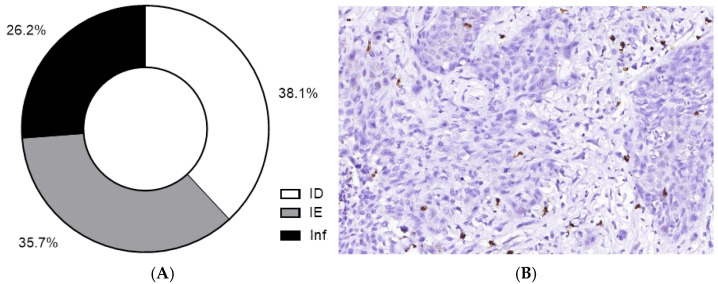

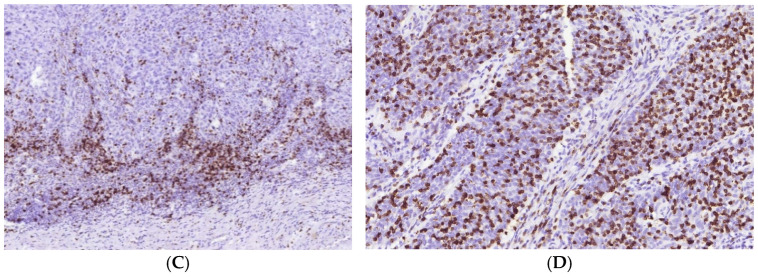
The heterogeneity of tumor immune contexture in MIUC. The proportion of various tumor immune microenvironment types (**A**). The majority of MIUCs demonstrated immune-desert (**B**) or immune-excluded TIME (**C**). Only about a quarter of the MIUCs yielded a high number of CD8+ lymphocytes typical for inflamed TIME (**D**). (**B**,**D**) Immunohistochemistry with antibodies against CD8. Magnification: (**B**) 200, (**C**) 100, (**D**) 200.

Overall, ID and IE TIME prevailed among the observed cases of MIUC, reflecting the depletion of CD8+ lymphocytes’ activation or recruitment into tumors. Most MIUCs with a papillary structure had the IE-phenotype, with lymphocytes’ accumulation around the invasive margin of the tumor (Table 5). MIUC with conventional histology varied in TIME, while areas of squamous cell differentiation demonstrated the ID or IE immunophenotype (Figure 6). Inflammatory TIME was mostly associated with the basaloid or the sarcomatoid subtypes. 

An assessment of TIME’s impact on patients’ prognosis did not uncover a significant impact on patients’ outcome (Table 2). The number of CD8+ cells also did not differ between survivors and patients with poor outcomes. At the same time, the unfavorable outcome was associated with profoundly higher CD163+ cells in both tumor clusters and stroma (Table 5). 

### 3.3. Immunophenotype and PD-L1 Expression

**Figure 7:**
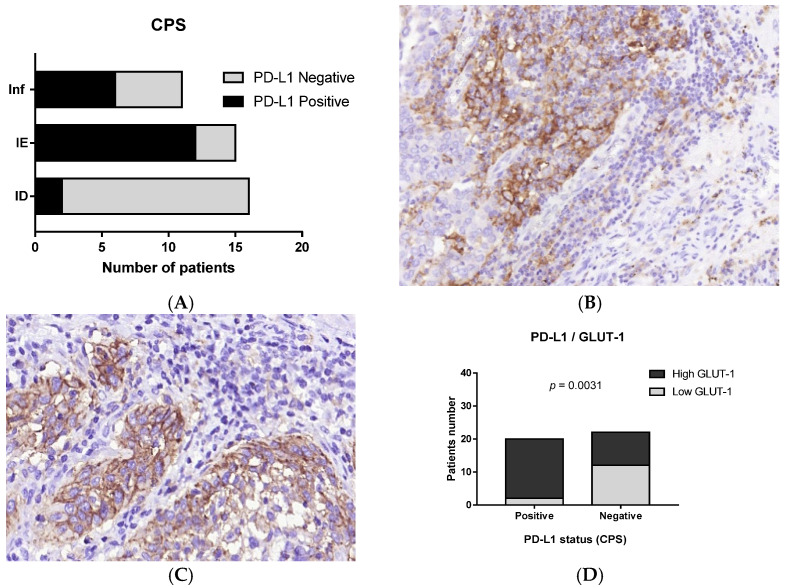
PD-L1 expression in MIUCs. PD-L1 rate in MIUCs of different TIME (**A**). PD-L was expressed in both tumor and immune cells (**B**,**C**) and was associated with the high expression of GLUT-1. (**A**) Schematic diagram representing the shares of PD-L1-positive and negative cases with respect to tumor immune microenvironment type. (**B**,**C**) Immunohistochemistry, using antibodies against PD-L1. Magnification: (**B**) 100, (**C**) 400. (**D**) Chart demonstrating the link between PD-L1 status and GLUT-1 expression. High GLUT-1 expression was associated with PD-L1-positive status.

There were 20 (47.6%) PD-L1-positive MIUCs assessed by CPS. PD-L1 expression was found both in tumor and immune cells, predominating in macrophages. Despite the lack of a relationship between PD-L1 expression and TIME type, most PD-L1-positive cases (14 out of 20; 70%) were of the IE- or ID-immunophenotype, while only 6 (30%) PD-L1-positive carcinomas possessed Inf TIME (Figure 7). Interestingly, in IE cases, PD-L1 expression was found predominantly at the invasive margin rather than in the central areas of the tumor. We failed to find a relationship between PD-L1 expression and histological differentiation.

**Figure 8:**
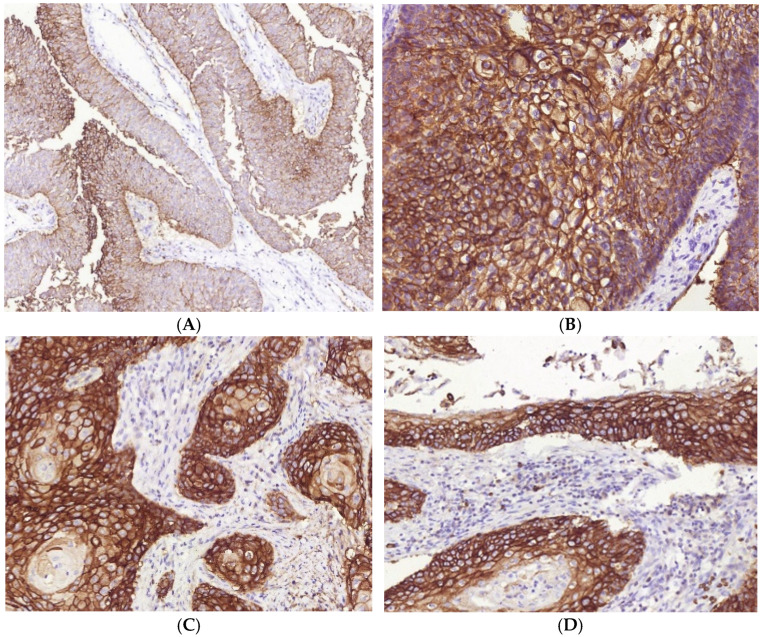
Peculiarities of GLUT-1 expression in MIUCs of various histological types. MIUCs with papillary patterns demonstrated low GLUT1 expression in exophyte portions (**A**) as compared to conventional invasive parts (**B**). The highest score of GLUT1 expression was found in areas of squamous differentiation (**C**). High expression of the hypoxic marker was also revealed in CIS (**D**). Immunohistochemistry with antibodies against GLUT1. Magnification: (**A**) 100, (**B**–**D**) 200.

In contrast to other cancer types, we did not reveal the relationship between PD-L1 expression and CD8+ cells or M2-macrophages numbers. However, PD-L1 was linked to GLUT1 expression (Figure 7). Although GLUT1 expression was found among all observed cases, it was higher in the areas of squamous differentiation as compared to MIUCs with the papillary pattern, though other histological types did not differ significantly in GLUT1 expression (Figure 8). Moreover, CD163+ cells and the PMN-infiltration range were related to the GLUT1 expression score.

### 3.4. Immune Cells and Hypoxia in MIUCs

**Figure 9:**
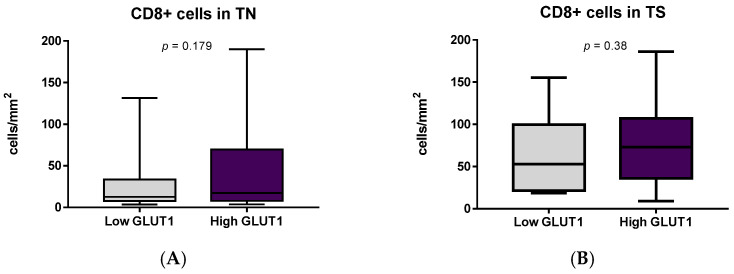

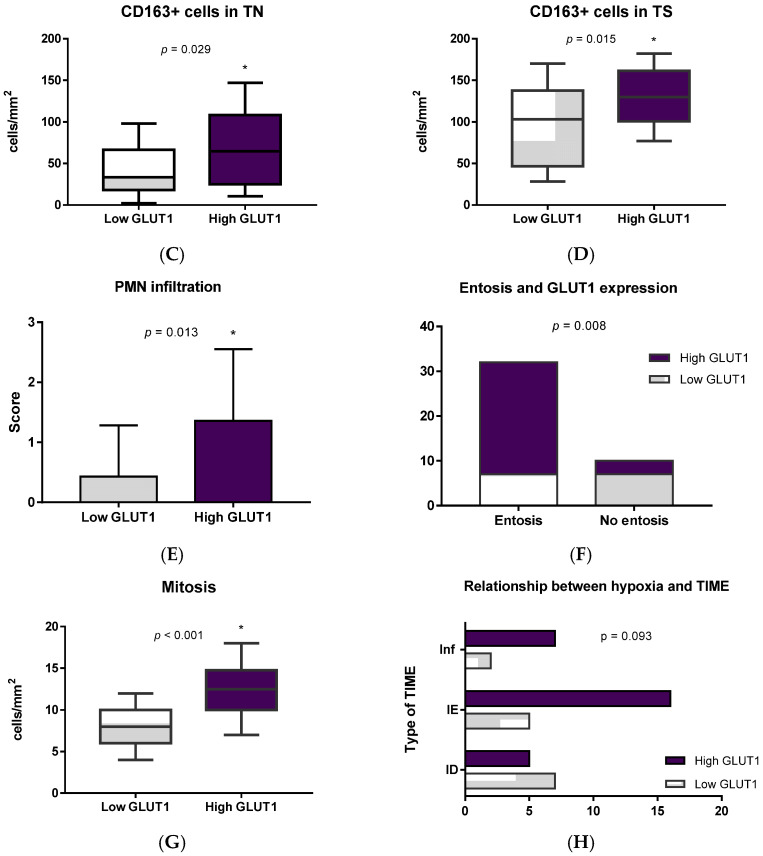
Relationship between immune cell number and hypoxic marker expression. Number of CD8+ cells number in tumor nests and stroma did not differ in tumors with various GLUT1 scores (**A**,**B**), though CD163+ cells (**C**,**D**) and PMN leukocytes (**E**) were more numerous in carcinomas with high GLUT1 expression. Besides, hypoxic conditions were associated with moderate or severe entosis (**F**) and higher mitosis rate (**G**), but not TIME type (**H**).

Assessment of immune cells in MIUC depending on the GLUT1 score elucidated the effect of hypoxia on CD163 and PMN cell numbers. Both neutrophils and M2-macrophages prevailed in MIUCs with high GLUT1 scores, though there were no differences in number of CD8+ cells with respect to GLUT1 expression (Figure 9). 

Addressing tumor biological features, we also found the link between hypoxia and mitosis number (*p* = 0.001). Naturally, tumor cell proliferation was high in MIUCs with prominent GLUT1 expression. Besides, entosis pictures were closely associated with prolonged hypoxia. Entosis was almost observed in carcinomas with dense infiltration by macrophages and PMN cells.

### 3.5. Prognostic Value of the Immune Cells, PD-L1 Expression and GLUT1

**Figure 10:**
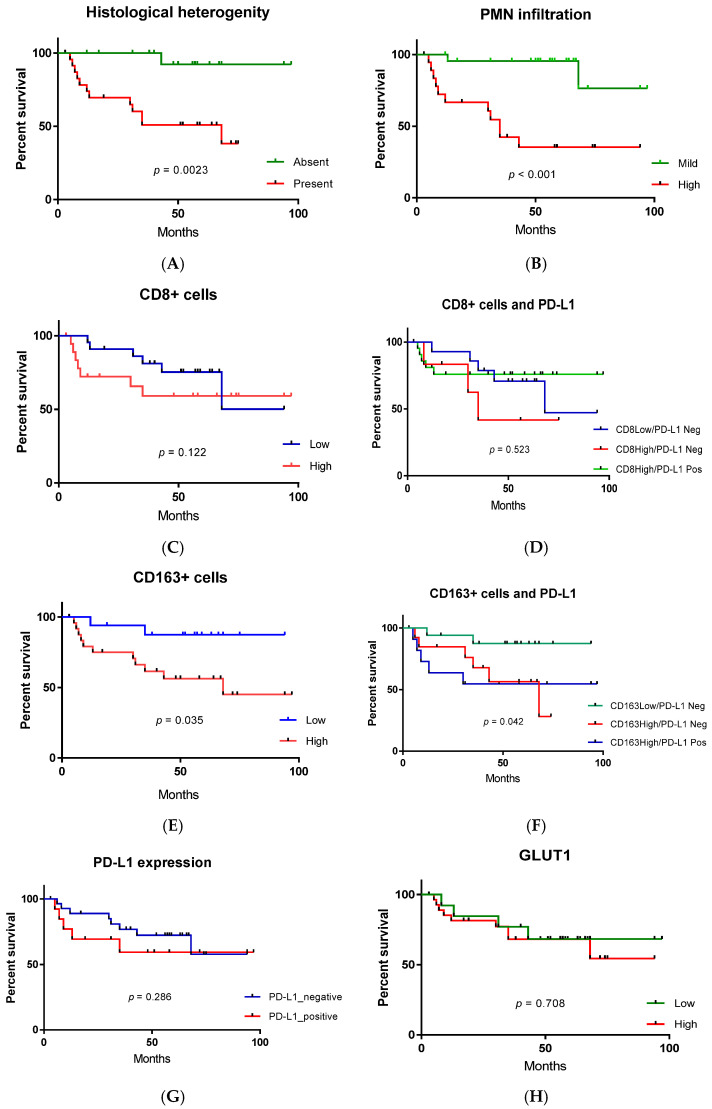
Kaplan–Meier curves for estimating factors’ impacts on survival in patients with MIUC. The effects of tumor variant histology (**A**), PMN infiltration (**B**), CD8+ (**C**) and CD163+ cells (**E**), PD-L1 (**G**) and GLUT1 (**H**) on patients’ survival were evaluated. CD8+ cells’ density, PD-L1 and GLUT1 expression did not affect the outcome in MIUCs. CD8 and PD-L1 combinations (**D**) also did not affect patients’ survival. Variant histology, high CD163 (independent of PD-L1 (**F**)) and PMN infiltration undermined patients’ survival.

For assessing the prognostic significance of immune cells, patients’ survival was assessed with respect to immune parameters. For this aim, we dichotomized tumors into distinct categories of CD8_high_ and CD8_low_ or CD163_high_ and CD163_low_ tumors. Depending on the PD-L1 expression, we assessed the tumor immune features depending on CD8/PD-L1 and CD163/PD-L1 status combinations (Figure 10). 

There was no impact of CD8+_high/low_ status on the survival of patients with MIUCs among the observed cohort, independently of PD-L1 status. At the same time, CD163_high_ status was associated with reduced survival (*p* = 0.014) in both PD-L1-positive and negative cases.

To note, variant histology and prominent PMN infiltration were associated with the decline in patients’ survival and unfavorable outcomes (*p* < 0.001).

## 4. Discussion

The results of the study revealed that histological differentiation defines the MIUC immunogenicity affecting both innate (PMN leucocytes and CD163+ macrophages) and adaptive immunity (CD8+ cells) cells. In contrast to common findings, we established significant differences of immunogenicity between squamous and basaloid differentiation of urothelial carcinomas. While tumor clusters with squamous differentiation demonstrated scant CD8+ cells and intense PMN infiltration, basaloid differentiation yielded a high number of CD8+ cells. Besides, the papillary pattern of differentiation was associated with low CD8+ and PMN infiltration. Therefore, various histological patterns demonstrated an association with predominant activation of immune mechanisms. Interestingly, the same phenomenon was previously observed in gastric carcinoma, in which histological differentiation defined the tumor immunogenicity [15]. The relationship between tumor-infiltrating immune cells and histological types of MIUCs was also shown in Kim et al.’s study [3]. Although some studies demonstrated basal and squamous markers’ overlap related to the common root of cell lineage, morphologically squamous features did not always match with the basal signature and the basal group tumors did not always possess squamous features [9]. Diverse immunogenicity of MIUCs was the most obvious in MIUCs with structural heterogeneity, demonstrating differences in both clonal histological differentiation and respective immunogenicity.

The most intriguing question is why urothelial carcinomas of various histological types differ in immunogenicity. There is no clear understanding of this issue, however, there are some assumptions that various lineages of cancer cells possess different immunomodulating factors, affecting the tumor–host interplay [10]. Assessing tumors with heterogeneous histological features provided a clear understanding that lineage-specific differentiation can determine tumor immunogenicity. Heterogeneity of MIUCs can be caused by different cancer stem cells and their clones, resulting in a mixture of signatures and discordance between global mRNA profiling and immunohistochemical features that is relevant not only for tumor cells but also for immune cells and their signatures. Besides, DNA damage can trigger innate immune responses through the accumulation of single-stranded and double-stranded DNA in the cytoplasm and their release into the extracellular environment [11]. Alterations in DNA-damage response machinery can heavily affect MIUC behavior, defining molecular features of MIUCs, their inflammatory pathways and immune contexture through generated neoantigens [39].

Sjödahl et al. illuminated significant differences in the number of TILs and TAMs in MIUCs of various molecular subtypes, which also demonstrated distinct histological features [7]. Therefore, differences in CD8+ infiltration could be related to variations of genomic instability in MIUCs of certain histological and molecular subtypes. According to the international consensus, a set of six molecular classes was identified, including: luminal papillary (24%), luminal non-specified (8%), luminal unstable (15%), stroma-rich (15%), basal/squamous (35%) and neuroendocrine-like (3%) [7]. These classes vary in molecular oncogenic mechanisms, harbored mutations and infiltration by immune and stromal cells. 

Despite the advances in discovering the molecular landscape of MIUC, there is a strong limitation of clustering MIUCs due to tumor heterogeneity that appears not only histologically but also at the level of the genes and proteins expressed [6]. Besides, previous studies showed a pseudo-differentiation phenomenon when the expression of urothelial differentiation factors was inconsistent with the protein expression of terminal differentiation markers [8]. Therefore, evaluating histological and immunogenic heterogeneity of MIUCs could be an important preliminary step for further molecular assessment.

Unlike other cancers, including colorectal and breast carcinoma, MIUCs did not demonstrate a link between the number of CD8+ cells and the prognosis of patients in this study. Instead, the role of TAMs was shown. The high prevalence of M2-macrophages in MIUCs and their predominance under T-cells could be one of the immune-mediated mechanisms of MIUC aggressiveness. A high TAM density has been reported to be associated with poor prognosis in various malignancies, such as breast cancer, melanoma, colorectal cancer and gastric and laryngopharyngeal cancer [24]. TAMs are well-known facilitators of tumor progression due to numerous growth factors and cytokines that promote tumor cell proliferation, invasiveness and angiogenesis [40], and immunosuppressive factors such as TGF-β, arginase 1, indoleamine 2,3-dioxygenase (IDO), IL-10 and programmed death ligand 1 (PD-L1). Besides M2-macrophages, derived factors are essential for the induction of T-regulatory cells (iTregs) which inhibit the adaptive immune response [40].

Despite the lack of a relationship between the number of CD163+ cells and PD-L1 expression, there was a link between PD-L1 status and GLUT-1 expression that could reflect the role of hypoxia in immune escape mechanisms. High levels of GLUT-1 expression in MIUC in fact reflect the hypoxia and hypoxia-associated metabolic alterations associated with glycolytic pathways’ activation (Warburg effect), facilitating cancer progression and predefining poor prognosis [28,41]. 

Notably, a high percentage of MIUCs in this study demonstrated PD-L1 expression in both tumor and immune cells. A study of urothelial cancer variants and PD-L1 expression demonstrated high tumor cell staining in squamous differentiation when compared to other variants [42]. Previous transcriptomic studies conducted on TCGA cohorts demonstrated that basal-type MIUCs referring to Clusters III and IV possessed high expression of MHC class II genes and IFN gamma-related genes, which indicates the stronger immunogenic nature of basal subtypes of MIUC [43]. Here, we found only the spatial relationship between PD-L1 expression and CD8+ cells’ accumulation at the invasive margin of MIUCs that could reflect one of the mechanisms of immune escape. 

Although the underlying mechanisms of PD-L1 expression in cancer are not completely established, there are some data confirming the role of M2-macrophages in PD-L1 expression. Some of the signaling pathways of such link are related with activation of COX-2 and prostaglandin E_2_ (PGE_2_) synthesis in macrophages, which connects inflammatory-mediated tumor remodeling and can induce proinflammatory activation of PMNs [44].

Considering the impact of TAMs and PMNs on the OS of patients with MIUC, it seems that innate immunity cells’ assessment could be a promising complementary biomarker for prognostication in MIUC; however, its reliability should be confirmed in further prospective studies. The shift from an adaptive to an innate immunity reaction with the prevalence of M2-macrophages reflects the dysregulation of the immune microenvironment that can contribute to MIUC progression and adverse outcomes. Additionally, a high rate of PD-L1 expression linked to hypoxia could be an additional immune escape mechanism.

## 5. Conclusions

MIUC demonstrated the suppression of cancer-antagonizing cells, though their immune contexture was tightly related to histological variability. The abundance of PMN and CD163+ macrophages in MIUC was associated with high GLUT1 expression and negatively impacted patients’ outcomes, independently of CD8+ cells’ infiltration and PD-L1 expression. Although most MIUCs yielded immune-desert or immune-excluded TIME, neither the number of CD8+ cells nor PD-L1 status affected prognosis in MIUC.

These data drive the hypothesis that in MIUC, immune escape mechanisms are shifted towards the role of the suppressive innate immunity cells rather than CD8+ lymphocytes’ functioning.

## Figures and Tables

**Table 1:** Antibody sources and interpretation.

Markers	Main Target	Antibody Characteristics	Cellular Localization	Rationale
CD8	T-cell	Dako; Clone C8/144B	Membranous	The CD8 antigen is found on most cytotoxic T-lymphocytes and acts primarily as a coreceptor for MHC class I molecule. CD8 contributes to efficient cell–cell interactions in antitumor immunity.
FoxP3	T-cell	Cell Marque, Clone EP340	Nuclear	FOXP3 is a member of the forkhead/winged-helix family of transcriptional regulators that is crucial for the development and suppressive function of T-regulatory cells (Treg) [32].
CD68	Macrophage	Dako, Clone KP1	Cytoplasmic	CD68 is considered a pan-macrophage marker. It is a member of the lysosomal/endosomal-associated membrane glycoprotein localized to lysosomes and plays a role in the phagocytic activities of tissue macrophages. CD68 is also a member of the scavenger receptor family, acting to clear cellular debris and mediate the recruitment and activation of macrophages.
CD163	M2-macrophages	Cell Marque, Clone MRQ-26	Cytoplasmic	CD163 is a member of the scavenger receptor superfamily and a marker of alternatively activated or anti-inflammatory macrophages. CD163 is involved in the clearance of hemoglobin/haptoglobin complexes, contributing to tissue protection from free hemoglobin-mediated oxidative damage, and additionally acts as an innate immune sensor. Its role was shown in defining bladder cancer prognosis and sensitivity to therapy.
PD-L1	Immune checkpoint	Dako, 22C3	Membranous	PD-L1 (CD274) is a ligand that binds with the PD1 receptor, reducing T-cell effector response and cytokine production, providing an immune escape for tumor cells [33] and facilitating tumor survival [34].
GLUT1	Hypoxic conditions	Cell Marque, polyclonal	Membranous	GLUT1 is energy-independent glucose transporter 1, or Solute Carrier Family 2 Member 1 (SLC2A1), with a high affinity for glucose. There is no GLUT1 expression in normal bladder mucosa, however it is upregulated in urothelial carcinomas [35] and is considered to be a metabolic marker of prolonged hypoxia [36].

**Table 2:** Baseline patients’ characteristics.

Characteristics	Value	%
Total number of the enrolled patients	42	100
Men	42	100
Age	56.8 ± 1.7 (53.4–60.2)	
Under 55 years	13	30.9
Over 55 years	29	69.1
PFS	20	47.6
Survived	29	69
Died	13	31
Pathological features		
pT staging		
pT2	30	71.4
pT3	12	28.6
CIS presence	14	33.3
Lymphovascular invasion	22	52.4
Variant histology	26	61.9
TIME type		
ID	16	38.1
IE	15	35.7
Inflamed	11	26.2

Data are presented as *n* (%) or mean ± standard deviation (SD) with 95% confidence interval (CI).

**Table 3:** The effect of different factors on the 5-year survival rate in MIUC.

	Hazard Ratio	95% CI	*p*
pT3	3.91	0.96–10.14	0.049
LVI	6.19	2.06–18.56	0.0017
CIS	5.48	1.838–16.34	0.0024
Variant histology	8.295	2.437–28.23	0.0023
TLS	0.487	0.1597–1.485	0.242
TIME ID and IE vs. Inf	0.57	0.1874–1.762	0.295
CD8+	1.74	0.5675–5.336	0.122
CD68+	1.42	0.439–4.632	0.338
CD163+	3.388	1.13–10.16	0.035
PMN infiltration	8.358	2.675–26.12	0.001
PD-L1	1.544	0.466–5.117	0.286
GLUT1 low/high	1.243	0.398–3.882	0.708

**Table 4:** Histological subtypes of MIUCs among the observed cohort.

Histological Subtype	Number of Cases	%
Papillary-invasive carcinoma	9	21%
Urothelial-invasive carcinoma (UIC)	7	17%
UIC + squamous differentiation	14	33%
UIC + micropapillary differentiation	2	5%
UIC + poorly differentiated (basaloid)	2	5%
UIC + sarcomatoid (mesenchymal-like)	4	10%
UIC + 2 and more differential patterns	4	10%

**Table 5:** Comparison of immune microenvironment parameters between patients with different outcomes.

	Outcome		
	Good Prognosis	Poor Prognosis	
	Mean ± SEM 95% CI	Mean ± SEM 95% CI	*p*
ID	10	6	0.4930
IE	10	5	
Inf	9	2	
CD8+ cells in tumor clusters	30.2 ± 6.42 17.1–43.4	40.1 ± 15.0 7.4–72.8	0.479
CD8+ cells in peritumoral stroma	78.1 ± 10.22 57.1–98.9	79.7 ± 15.7 45.6–113.9	0.929
CD163+ cells in tumor clusters	44.5 ± 6.40 31.4–57.7	98.4 ± 10.7 74.9–121.8	<0.001
CD163+ cells in tumor stroma	102.7 ± 8.45 85.3–120.0	156.7 ± 14.1 125.9–187.6	0.001

## Data Availability

The data that support the findings of this study are available upon request from the corresponding author.

## Limitations of the Study

This study was performed on a limited number of males with MIUC of pT2–3 stages. The lack of molecular testing data could also be considered as a limitation for extrapolation on the molecular classification of MIUCs and possible intrinsic mechanisms of carcinogenesis.
